# Inhibitory effects of garcinone E on fatty acid synthase

**DOI:** 10.1039/c7ra13246h

**Published:** 2018-02-20

**Authors:** Yan Liang, Di Luo, Xuan Gao, Hao Wu

**Affiliations:** School of Kinesiology and Health, Capital University of Physical Education and Sports Beijing 100191 China yanliang@cupes.edu.cn gaoxuan713@cupes.edu.cn +86-10-82099150 +86-10-82099150; Scientific Research Office, Capital University of Physical Education and Sports Beijing 100191 China luodi@cupes.edu.cn wuhao@cupes.edu.cn +86-10-82099150 +86-10-82099150

## Abstract

Fatty acid synthase (FAS) is highly expressed in human adipocytes and cancer cells and is considered as a dual therapeutic target for obesity and cancer treatment. Garcinone E is a natural xanthone and exists in the pericarp of *Garcinia mangostana*. In previous studies, xanthones were reported to be highly active inhibitors of FAS. In the present study, the detailed inhibitory mechanism of garcinone E on FAS was investigated. We found that garcinone E inhibited the activity of FAS in a concentration-dependent manner with a half-inhibitory concentration value of 3.3 μM. The inhibition kinetic results showed that the inhibition of FAS by garcinone E was competitive with respect to acetyl-CoA, mixed competitive and noncompetitive with respect to malonyl-CoA, and noncompetitive to NADPH. In addition, garcinone E showed irreversible inhibition on FAS, which was different from all other xanthones. Since FAS is believed to be a therapeutic target for obesity and cancer treatment, these findings suggest the clinical potential of garcinone E in the prevention and treatment of both obesity and cancer.

## Abstract 2

Garcinone E exhibits both fast-binding reversible and time-dependent irreversible inhibition on the activity of fatty acid synthase.

## Graphical abstract

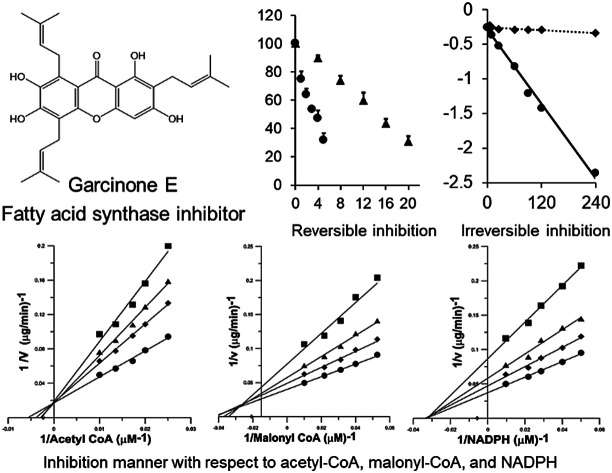

## 1. Introduction

Fatty acid synthase (FAS, EC 2.3.1.85) catalyzes the synthesis of saturated long-chain fatty acids, predominately palmitate, from acetyl-CoA (Ac-CoA) and malonyl-CoA (Mal-CoA) precursors, in the presence of NADPH.^1–3^ As a multifunctional enzyme complex, FAS comprises two identical subunits, each containing seven discrete functional domains: malonyl/acetyltransferase (MAT), β-ketoacyl synthase (KS), β-ketoacyl reductase (KR), dehydrase (DH), enoyl reductase (ER), thioesterase (TE), and the acyl carrier protein (ACP).^4^ Inside the human body, most normal tissues exhibit low levels of FAS expression. However, human adipocytes and cancer cells express high levels of FAS and undergo significant endogenous fatty acid synthesis.^5,6^ In adipocytes and cancer cells, the functional connection between FAS and cell proliferation, lipid deposition, progression, aggressiveness, and metastasis has been provided by the discovery and design of small molecule drugs that inhibit the catalytic activity of FAS.^7–10^ Previous studies have shown that FAS inhibitors such as C75, cerulenin and epigallocatechin gallate (EGCG) lead to weight loss, preadipocyte proliferation inhibition, and cancer cell apoptosis.^11–13^ Therefore, FAS deemed as a potential bi-target for anti-obesity and anti-cancer drugs.^14–17^

Garcinone E (2,3,6,8-tetrahydroxy-1,4,7-tris(3-methylbut-2-enyl)-9*H*-xanthen-9-one) is a xanthone which exists in the pericarp of *Garcinia mangostana*.^18^ Experimental results have demonstrated that the xanthones isolated from *G. mangostana* exhibit antioxidant, antitumoral, antiallergic, anti-inflammatory, antibacterial, and antiviral activities.^19–21^ However, the literature related to the application of garcinone E in prevention and treatment of obesity or cancer is limited. It was reported that garcinone E induced significant cell cycle arrest at G0/G1 phase in hepatocellular carcinoma cell lines, as well induced apoptosis and inhibited migration and invasion in ovarian cancer cells.^22,23^ Nevertheless, the precise mechanisms involved are still not demonstrated clearly.

In previous studies, garcinone E, as well as other xanthones isolated from the pericarp of *G. mangostana*, has been found strong inhibitory activity on FAS overall reaction. Compared with the wellknown FAS inhibitors such as C75 and EGCG, garcinone E showed stronger inhibitory activity.^18^ In the present study, we further investigated the inhibitory kinetics of garcinone E on FAS activity.

## 2. Materials and methods

### 2.1 Reagents

Garcinone E (98% HPLC), Ac-CoA, Mal-CoA, NADPH, ethyl acetoacetate, ethylenediaminetetraacetic acid (EDTA), dithiothreitol (DTT), and dimethyl sulfoxide (DMSO) were purchased from Sigma-Aldrich. Other reagents were of analytical grade.

### 2.2 Preparation of FAS and its substrates

The preparation of FAS from chicken liver (Huadu Broiler Corporation, Beijing) was performed as described previously.^24^ The amino acid sequence of chicken FAS has 63% identity with the sequence of human enzyme.^25^ All animal operations followed the Guidelines for the Care and Use of Laboratory Animals established by the National Research Council (US) Institute for Laboratory Animal Research. The purified FAS was homogenized by polyacrylamide gel electrophoresis in the presence and absence of sodium dodecyl sulfate, respectively. The concentrations of FAS and its substrates were determined by UV-vis spectrophotometer (Amersham Pharmacia Ultrospec 4300, England, UK) using the following experimental parameters: FAS, 4.83 × 10^5^ M^−1^ cm^−1^ at 279 nm; Ac-CoA, 1.54 × 10^4^ M^−1^ cm^−1^ at 259 nm, pH 7.0; Mal-CoA, 1.46 × 10^4^ M^−1^ cm^−1^ at 260 nm, pH 6.0; acetoacetyl-CoA, 1.59 × 10^4^ M^−1^ cm^−1^ at 259 nm, pH 7.0; NADPH, 6.02 × 10^3^ M^−1^ cm^−1^ at 340 nm, and 1.59 × 10^4^ M^−1^ cm^−1^ at 259 nm, pH 9.0 ^26^.

### 2.3 Assays of FAS activity

The FAS activity was measured at 37 °C by spectrophotometer at 340 nm of NADPH absorption. The overall reaction system contained 100 mM KH_2_PO_4_–K_2_HPO_4_ buffer, 1 mM EDTA, 1 mM dithiothreitol, 3 μM Ac-CoA, 10 μM Mal-CoA, 35 μM NADPH, and 10 μg FAS in a total volume of 2 ml as previously described.^26,27^ The assay for β-ketoacyl reduction activity of FAS was catalyzed by KR containing 100 mM KH_2_PO_4_–K_2_HPO_4_ buffer, 200 mM ethyl acetoacetate, 35 μM NADPH, and 20 μg FAS in a total volume of 2 ml (pH 7.0).

### 2.4 Assays of FAS inhibition

Reversible inhibition was determined by adding the inhibitor to the reaction system before the reaction was initiated. The activities of FAS in the presence and absence of garcinone E (dissolved in DMSO) were designated as *A*_i_ and *A*_0_ respectively. The value of *A*_i_/*A*_0_ × 100% was defined as the relative activity (R.A.) of FAS. The half-inhibitory concentration value (IC_50_) was obtained from a plot of R.A. *versus* garcinone E concentration. In the experiments the largest volume added to the reaction was far less than 0.5% (v/v); thus the DMSO existing in the reaction system had no influence on the results of the activity assay.

The activity of KR was determined at 37 °C by measuring the change of absorption at 340 nm. The KR reaction mixture (2 ml) contained 40 mM ethyl acetoacetate, 35 μM NADPH, 1 mM EDTA, 1 mM DTT and 10 μg of FAS in 100 mM phosphate buffer, pH 7.0.^28^

Assay of time-dependent inhibition activity was carried out as previous described.^13^ The FAS solution was mixed with garcinone E and incubated at 25 °C, and then aliquots were taken to measure the remaining activity at the indicated time intervals to obtain the time course. This time-dependent inhibition is usually caused by a chemical combination of the inhibitor with the enzyme, and is irreversible. The rate constant of FAS inactivation can be calculated from a semi-log plot of the time course, which is based upon the formula ln *A*_*t*_/*A*_0_ = −*k*_obs_*t*. The *A*_*t*_/*A*_0_ expresses the remaining activity at *t* time, and *k*_obs_ is the observed rate constant. Based on earlier studies, the inhibition of FAS activity is due to both fast-binding and time-dependent inhibitions, although sometimes the fast-binding reversible inhibition is not potent enough to affect the enzyme.

### 2.5 Enzyme kinetics study

Possible interference by the inhibitor at each substrate binding site was examined by holding the concentration of the inhibitor at several fixed levels respectively, and increasing one substrate concentration while keeping the concentrations of the other substrates constant. Double reciprocal plots for every concentration of the inhibitor were yielded to estimate the competitive relationship between the variable substrate and inhibitor concentrations. This study is based on fast-binding inhibition.

## 3. Results

### 3.1 Inhibitory effect of garcinone E on FAS overall and β-ketoacyl reduction activities

**Fig. 1:**
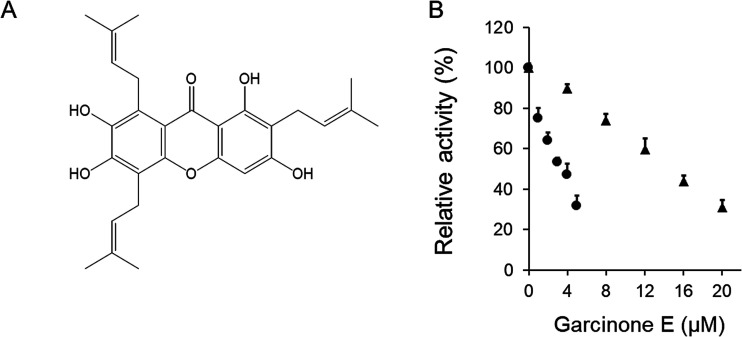
The fast-binding reversible inhibitory effect of garcinone E on FAS overall and β-ketoacyl reduction activities. (A) The chemical structure of garcinone E. (B) The overall reaction (●) and KR reaction (▲) of FAS were measured in the presence of various concentrations of garcinone E. Values represented the mean ± SD of triplicate determinations.

The inhibitory activity of garcinone E on overall reaction and KR of FAS was assayed *in vitro*. Garcinone E exhibited strong fast-binding inhibition of chicken FAS. The data obtained from Fig. 1B showed that 3.3 μM of garcinone E inhibited 50% of the overall reaction activity and 14.6 μM of garcinone E inhibited 50% of the KR activity.

### 3.2 Irreversible inhibitory effect of garcinone E on FAS

**Fig. 2:**
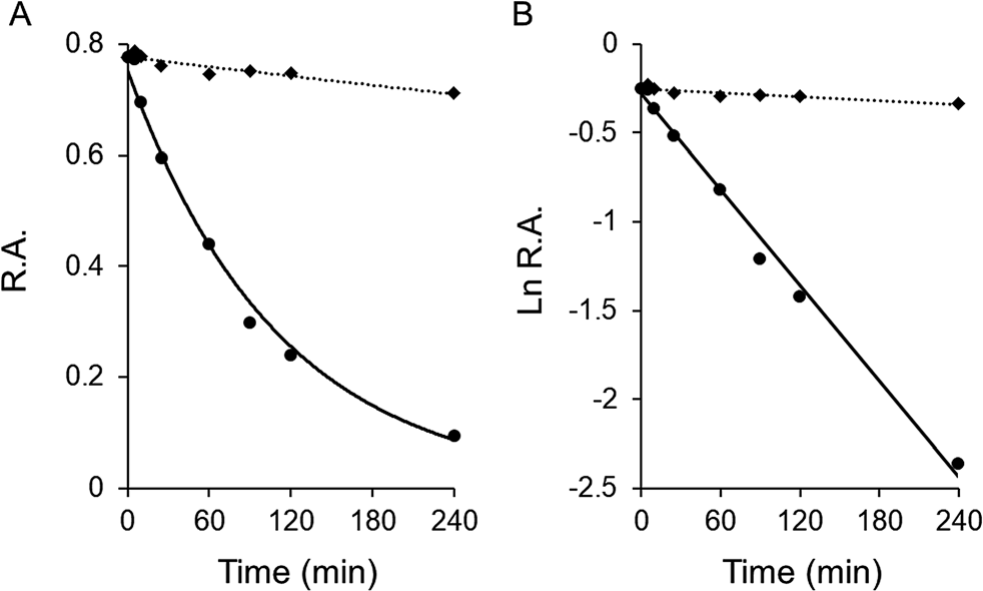
The time-dependent irreversible inhibitory effect of garcinone E on FAS activity. The time-dependent inhibition of the overall reaction and KR of FAS was measured in the presence of garcinone E. (A) The FAS solution was mixed with 20.0 μM garcinone E, and aliquots were taken and assayed for relative activity at the indicated time intervals. (R.A. = relative activity) (B) semi logarithmic diagram of time and R.A.

Garcinone E exhibited time-dependent inhibition of FAS overall reaction. Fig. 2A showed the time-course slow-binding inhibition of garcinone E on overall reaction and KR of FAS. Garcinone E exhibited a slow-binding irreversible inhibition on overall reaction of FAS. However, the irreversible inhibition of garcinone E on KR was very weak. The rate constants of FAS inactivation were calculated from the semi logarithmic diagram of time and relative activity (as shown in Fig. 2B). With a garcinone E concentration of 20.0 μM, *k*_obs_ values were 0.009, and 0.0004 min^−1^ for overall reaction and KR, respectively. The more than 20-fold difference in the inactivation rate between overall reaction and KR suggested that the irreversible inhibition of garcinone E should be rarely related to KR activity.

### 3.3 Kinetic mechanism of inhibition of FAS by garcinone E

**Fig. 3:**
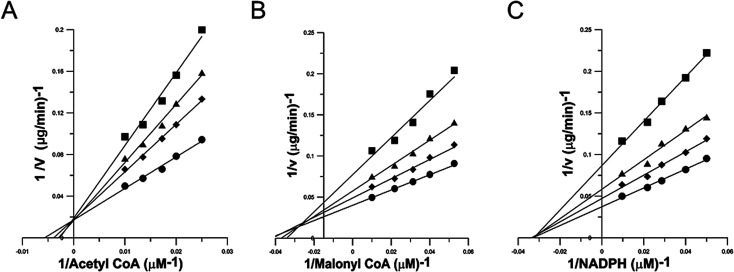
Lineweaver–Burke plots for inhibition of FAS activity by garcinone E. Double reciprocal plots for inhibition of FAS by garcinone E. The activity of the overall reaction of FAS was measured. (A) The concentrations of Mal-CoA and NADPH were fixed at 10 μM and 35 μM, respectively. Ac-CoA was the variable substrate. The concentrations of garcinone E were: 0 μg ml^−1^ (●), 0.7 μg ml^−1^ (◆), 1.3 μg ml^−1^ (▲), and 2.2 μg ml^−1^ (■). (B) The concentrations of Ac-CoA and NADPH were fixed at 3 μM and 35 μM, respectively. Mal-CoA was the variable substrate. The concentrations of garcinone E were: 0 μg ml^−1^ (●), 0.5 μg ml^−1^ (◆), 1.7 μg ml^−1^ (▲), and 3.3 μg ml^−1^ (■). (C) The concentrations of Ac-CoA and Mal-CoA were fixed at 3 μM and 10 μM, respectively. NADPH was the variable substrate. The concentrations of garcinone E were: 0 μg ml^−1^ (●), 0.4 μg ml^−1^ (◆), 1.2 μg ml^−1^ (▲), and 2.5 μg ml^−1^ (■). Each datum was the mean from 2–5 experiments.

Inhibition kinetics study was carried out to elucidate the mechanism of the reversible inhibition of FAS by garcinone E. Possible interference by garcinone E at each substrate-binding site was examined by holding the concentration of garcinone E at a constant value, and measuring the effect of increasing one substrate concentration (the concentrations of the other two substrates were fixed) on the initial reaction rate. Double-reciprocal plots of the results (Fig. 3A–C) yielded three families of straight lines, which had different intersections, for three substrates of FAS. These lines for Ac-CoA (Fig. 3A) had a common intercept on the *Y* axis, indicating that garcinone E inhibited FAS competitively with Ac-CoA. The dissociation constant for inhibitor binding, *K*_is_ of 1.77 μM, was obtained from the secondary plot of the slopes of these lines *versus* garcinone E concentration. The lines for Mal-CoA (Fig. 3B) had a point of intersection in the second quadrant, indicating that the inhibition was of mixed competitive and noncompetitive type. The dissociation constant for inhibitor binding, *K*_is_ of 3.89 μM, was obtained from the secondary plot of the slopes of these lines *versus* garcinone E concentration. While the dissociation constant for garcinone E binding with enzyme-substrate complex (ES), *K*_ii_ of 5.99 μM, was obtained from the secondary plot of the intercepts *versus* garcinone E concentrations. However, the lines for NADPH (Fig. 3C) intersected on the *X* axis, from which it was concluded that garcinone E was a typically noncompetitive inhibitor of FAS against NADPH. The dissociation constant for inhibitor binding was calculated from the secondary plot of slopes *versus* garcinone E concentrations (*K*_is_ of 1.94 μM) or from the secondary plot of intercepts *versus* garcinone E concentrations (*K*_ii_ of 2.67 μM). The inhibition types and inhibition constants of FAS for every substrate by garcinone E were summarized in Table 1.

**Table 1:** Inhibition types and inhibition constants of FAS for every substrate by garcinone E

Substrate	Inhibitory manner	Inhibition constant (μM)	
		*K*_is_	*K*_ii_
Ac-CoA	Competitive	1.77	—
Mal-CoA	Mixed competitive and noncompetitive	3.89	5.99
NADPH	Noncompetitive	1.94	2.67

Furthermore, substrate protection for time-dependent inhibition was examined in the measurement of FAS inactivation by garcinone E with 10 μM Ac-CoA, 10 μM Mal-CoA, or 10 μM NADPH, respectively. Inactivation in the same condition but without any substrate was the control. The result of substrate protection on the inactivation of FAS by 10.0 μM garcinone E was given in Table 2 and it was seen that the apparent rate constant was only reduced by less than 10% by pre-incubation with Mal-CoA and NADPH. Therefore, the substrates of Mal-CoA and NADPH had no obvious protection effect. However, the inactivation was slowed by Ac-CoA. The result suggested that this time-dependent inactivation may be related to Ac-CoA binding site on the KS and/or MAT domain.

**Table 2:** Rate constants for inactivation of FAS by garcinone E with protection by three substrates

Substrate for protection	No protection	Ac-CoA	Mal-CoA	NADPH
*k*_obs_ (10^−3^ min^−1^)	17.4	5.3	16.6	15.9

## 4. Discussion

In recent years, the morbidity of obesity or cancer is increasing at an epidemic rate. As a key enzyme which can regulate energy metabolism *in vivo*, FAS has been indicated as a feasible target for the treatment of both obesity and cancer. However, no anti-obesity and anti-cancer drugs have been developed from FAS inhibitors so far. It is necessary to discover more safe and effective FAS inhibitors that may be applied practically in treatment of obesity and cancer. The amino acid sequences of human and other animal FASs are very similar,^29^ however, the human FAS preparations showed lower activity than the FASs of other animals.^25^ So the chicken and duck FAS were the most commonly used materials for the study of FAS inhibitors. In this study, chicken FAS was used to measure the inhibitory activity as well as the inhibitory mechanism of garcinone E. In the present work, we found that garcinone E was a very potent FAS inhibitor with an IC_50_ value of 3.3 μM on the overall reaction of FAS, which was much lower than those of cerulenin (IC_50_ = 89 μM)^30^ and EGCG (IC_50_ = 52 μM).^13^ The strong inhibitory activity of garcinone E towards FAS, plus its edible plants source, opens up excellent prospects for its application as an anti-obesity and anti-cancer agent.

In order to demonstrate the inhibition mechanism of garcinone E on FAS, we further determined the kinetic parameters of FAS. We found that garcinone E could inhibit both FAS and KR domain. However, the IC_50_ value of β-ketoacyl reduction was 4.57 times higher than overall reaction. Garcinone E showed strong reversible inhibition on the overall reaction of FAS but showed weak inhibition on KR reaction. These results indicated that KR was not the main active site in FAS that attacked by garcinone E, which also was confirmed by kinetic results since garcinone E was a noncompetitive inhibitor of FAS with respect to NADPH.

As a wellknown FAS inhibitor, EGCG inhibits mainly the KR domain of FAS, and it binds to the same site competitively with NADPH. As for C75 and cerulenin, two other known FAS inhibitors, it has been reported that they inhibit FAS by irreversibly binding to the KS domain of FAS, and that their inhibition of FAS is related to the binding site of the substrate Mal-CoA. However, we found that garcinone E inhibited FAS in a mixed competitive and noncompetitive type with Mal-CoA. Therefore, as regards inhibition mechanism, garcinone E was totally different from the other FAS inhibitors previously reported. Garcinone E possibly affected the KS or the MAT domain of FAS because it inhibited FAS competitively with respect to Ac-CoA, Although the inhibitory activity of xanthones and other polyphenols on FAS was reported before, no slow-binding irreversible inhibition on FAS was found in these compounds. In the present work, we tested the time-dependent inhibitory effects of α-mangostin, β-mangostin, γ-mangostin, whose structures are similar to garcinone E, on FAS. However, none of them showed slow-binding inactivation towards FAS (data not shown). Since garcinone E comprises one more isoprene group compared with the structures of α-mangostin, β-mangostin, and γ-mangostin, the calculated lipid/water partition coefficient of garcinone E (7.9) was much bigger that those of α-mangostin (6.3), β-mangostin (6.6), and γ-mangostin (5.9). Maybe this could explain why garcinone E showed irreversible inhibition on FAS since it was easier to enter the interior of the FAS and combined with the active site of the enzyme because of the hydrophobic effect.

It has been reported that inhibition of FAS could selectively inhibit the growth and proliferation of human adipocytes and cancer cells because FAS activity is vital for the growth and survival of these cells. C75 could obviously reduce body weight and food intake in fat mouse.^11^ Cerulenin was found to inhibit adipocyte differentiation and lipid accumulation.^31^ α-Mangostin was reported to prevent human preadipocytes 3T3-L1 cells differentiation and reduce lipid accumulation as a potent FAS inhibitor. A number of studies have revealed the anti-obesity and anti-cancer effects of FAS inhibitors in human cancer cells and adipocytes.^32–34^

The potent anti-cancer effect of garcinone E was reported. However, the detailed mechanisms of apoptosis induction and invasion inhibitory effects of garcinone E on human cancer cells were not fully understood. We now speculate that garcinone E possibly performed anti-cancer activity, at least in part, by inhibiting FAS activity. Our present work not only demonstrated that garcinone E was a potent FAS inhibitor, but also revealed the possible mechanism of its inhibitory activity. Since garcinone E exists in edible plants, it might have great security for the human body and have promising application potential. This study may supply some useful idea and new clues in developing drugs in treatment of obesity and cancer.

## Author contribution statement

Yan Liang, Hao Wu as the principal investigators, were responsible for the concept and design of the study. Xuan Gao, Yan Liang, Di Luo did the whole experiments of the study, conducted the research and wrote the manuscript. All authors participated in the preparation of, and had approved the final version of the manuscript.

## Conflicts of interest

The authors confirm that this article content has no conflict of interest.

## Abbreviations

Ac-CoA: Acetyl CoA
ACP: Acyl carrier protein;
DH: Dehydrase
DMSO: Dimethyl sulfoxide
ER: Enoyl reductase
FAS: Fatty acid synthase
FBS: Fetal bovine serum
IC_50_: Half-inhibitory concentration
KR: Ketoacyl reductase
KS: Ketoacyl synthase
Mal-CoA: Malonyl-CoA
MAT: Malonyl/acetyltransferase
TE: Thioesterase

## Supplementary Material
